# Parental psychological control and adolescent social problems: The mediating effect of emotion regulation

**DOI:** 10.3389/fpsyt.2022.995211

**Published:** 2022-10-25

**Authors:** Guoying Qian, Yufeng Wu, Wei Wang, Lan Li, Xiaoyu Hu, Ruonan Li, Chunyu Liu, Ao Huang, Ruiqi Han, Yu An, Gang Dou

**Affiliations:** ^1^College of Preschool Education, Capital Normal University, Beijing, China; ^2^School of Psychology, Jiangxi Normal University, Nanchang, China; ^3^Research Center of Jiangxi Social Psychological Service System Construction, Nanchang, China; ^4^Jiangxi Key Laboratory of Psychology and Cognitive Science, Nanchang, China; ^5^Department of Psychology, Shanxi Datong University, Datong, China; ^6^Xi’an Gaoxin No. 1 Middle School, Xi’an, China; ^7^School of Education, Hubei University of Arts and Science, Xiangyang, China

**Keywords:** multi-child family, parental psychological control, emotion regulation, social problems, adolescent

## Abstract

This study aimed to investigate relationships among parental psychological control, adolescent emotion regulation, and social problems in China. In total, 1,145 adolescents aged 12–15 years participated in the study, which used the Parental Psychological Control Scale, Adolescent Problem Behavior Scale, and Emotion Regulation Scale. The results indicated the following: (1) Compared with only-child teens, adolescents in multi-child families had significant social problems; (2) parental psychological control significantly predicted adolescents’ social problems; (3) there was a partially mediating effect of adolescents’ emotion regulation between parental psychological control and adolescents’ social problems.

## Introduction

Many children in adolescence suffer from school weariness, depression, and social phobia (1, 2). Adolescence is a key stage of growth during which the individual undergoes a series of subtle changes in physiological, psychological, and social development, such as changes in hormone levels during puberty, continuous brain development, and the formation of individual independence. In this period, individual physical development is rapid, but psychological development is relatively slow. This imbalance in physical and mental development can easily lead to many psychological conflicts and contradictions, which often affects adolescents’ emotions and increases their need for emotion regulation (3). This increased need makes them use emotion regulation more frequently, and accordingly, the level of emotion regulation also determines the level of interpersonal interaction, problematic behavior, and mental health status of adolescents (1, 2).

The resource dilution theory (4) suggests that as the number of children in a family increases, the allocation of family resources for each child decreases, eventually resulting in a negative impact on parenting and more problem behaviors in the children. This phenomenon has been found in multi-child families (5).

Problem behavior refers to abnormal behavior that hinders individual social adaptation. In other words, it occurs when individuals violate or do not abide by social norms and codes of conduct or cannot adapt to social life well, thus causing a bad influence or even harm to society or themselves (6). Problem behaviors are classified into behaviors of social problems, thinking problems, and attention problems (7). Adolescents with social problems tend to have low self-esteem and lack necessary social skills. They often have unnecessary anxiety about social situations and interpersonal contact due to concerns about exposing their shortcomings or being negatively evaluated by others (2, 7). The formation of social problems is influenced by many factors. The problem behavior theory proposed by Jessor et al. explains the complex process of social problem formation in individuals to a certain extent (8, 9), which divides the factors into personality systems (individual’s attitude, values, personality, etc.) and situational system (school, family, peers, etc.) (2, 10). The problem behavior theory shows that family factors such as multi-child families, parental psychological control, and personality factors, such as an individual’s emotions, can affect adolescents’ social problems.

### Social problems of adolescents in multi-child families

Researchers have found that as the number of siblings in a family increases, the family resources allocated to each child decrease and the competition between siblings intensifies (4). Due to the newly revised family planning policy, the number of children in Chinese families is gradually increasing. Behavioral and emotional problems of siblings in these families are emerging, which has attracted increasing attention from social and academic researchers. Some studies have found that sibling jealousy and growing self-protection are associated with children withdrawing from their friendships or society, which in turn leads to many social problems (11). A study has found that boys in China’s multi-child families are more likely to engage in aggressive behavior and have discipline violations, while girls are more likely to have social problems such as social withdrawal (12). In China, parents of multi-child families typically experience more financial pressure than parents of one-child families. As they are often busy with work, they seldom have time and energy to take their children to participate in social activities, such as going to the movies, traveling, visiting relatives and friends, and so on (13). Especially in recent years, due to the COVID-19 pandemic, children have been busy either attending school or taking online classes at home, they are even more socially isolated than before, so they may have more social problems (14, 15).

### Relationship between parental psychological control and adolescent social problems

Parental psychological control belongs to parenting behavior, which refers to the behavior of parents violating adolescents’ emotional and psychological autonomy through verbal or non-verbal means in the process of parenting (16–20). Despite the fact that there are researches that underscored the importance of parental psychological control in the development and autonomy of Children (17), it remains a controversial dimension because of its complexity as a construct since, even though there is consensus about the negative association between parental psychological control and adolescent behavioral problems (21–24), the specific components of parental psychological control that contribute to preventing emotional and behavioral disorders are often not clear. In Barber et al. (25) made a progress in refining understanding of some of these components: Psychological control as manipulation and coercion, psychological control as intrusion into the personal domain and psychological control as disrespect. However, their finding remains to be further examined.

Adolescence is an important period for “separation-individuation.” Adolescents pursue autonomy and hope to get rid of parental authority and control (26, 27). The Self-Determination Theory states that individuals are born with a developmental tendency for self-growth and that the experience of parents meeting their children’s basic psychological needs, such as autonomy and competence, is key to achieving growth in potential (28, 29). However, parents with high psychological control often impose their own demands and wishes on their children by guiding their children to feel guilty, expressing their disappointment or ignoring and humiliating their children, which has a serious negative impact on the social development of adolescents (24, 28–30). One of the important indicators of social development is social behavior, and parental psychological control tends to cause low social connectedness in children, which, in turn, generates more social problems. For example, parental psychological control is positively correlated with social anxiety in adolescents (31); Shek (32) has argued that higher levels of parental psychological control are associated with relatively lower levels of trust and willingness to communicate with parents, which in turn increases the risk of aggressive behavior with others and affects their interactions with others, resulting in social problems.

### Mediating effect of emotion regulation

Emotion regulation is the process by which an individual influences the occurrence, experience, and expression of self-emotions, including cognitive reappraisal and inhibition of expression. Cognitive reappraisal refers to a process in which an individual changes their cognition and understanding of emotion-induced events and reconsiders their personal meaning. Inhibition of expression refers to the process by which an individual inhibits upcoming or ongoing emotional expression (33). Researchers generally agree that cognitive reappraisal is an adaptive emotion regulation strategy and expressive inhibition is a non-adaptive emotion regulation strategy. Cognitive reappraisal is better than expressive inhibition in regulating emotions and is beneficial to people’s physical and mental health (34, 35). Investigations show that any difficulty in emotion regulation may lead to social misconduct or social problems. In studies on adolescent emotion regulation and social development, adolescents with poor emotion regulation abilities lack social skills and show more problematic behaviors (36, 37). Adolescents’ choice of emotion regulation can predict individual social problems to a certain extent, and adolescents who choose positive emotion regulation have fewer social problems (38).

Morris et al. (39) have found that parenting style is associated with adolescents’ emotion regulation. Li et al. (40) also found that parental psychological control has an impact on the development of adolescents through emotion regulation. Adolescents with high psychological control have imperfect emotional and cognitive development, immature use of emotion regulation strategies, and are unable to actively deal with negative emotional experiences, resulting in emotional disorders. High levels of parental psychological control also tend to increase children’s negative emotions in daily interactions. When the level of parental psychological control increases, children’s negative emotional experience also increases. Parental psychological control negatively predicts adolescents’ emotional function (41). The more controlled adolescents are by their parents, the lower their emotion regulation ability will be (42).

**FIGURE 1:**
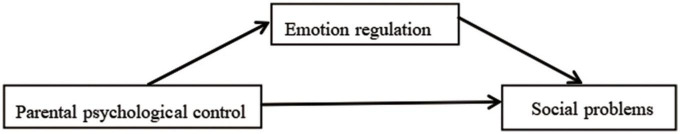
The proposed mediation model.

Based on the above research evidence and problem behavior theory, we hypothesize that emotion regulation plays a mediating role in parental psychological control and adolescents’ problem behavior in multi-child families (see Figure 1).

## Methods

### Participants

This study took a convenience sample of 1,488 adolescents in 7th and 8th grade in a school in Shaanxi Province, removed 343 people for incomplete completion of the questionnaire, and finally had a number of1,145, aged between 12 and 15, with 789 7th graders and 356 8th graders. There were 537 boys and 608 girls, 674 only children, 471 with siblings (including 41 who were the third child and above), 950 who were the oldest, 154 who were the second, and 41 who were the third and above. There were 268 people with a monthly family income of less than or equal to 5,000 yuan/month, accounting for 23.4%, 452 people with monthly family income between 5,001 and 10,000 yuan, accounting for 39.5%, and 425 people with monthly family income greater than 10,001yuan/month, accounting for 37.1%. Overall, 96.2% of the participant’s main caregivers were parents.

### Measures

#### Parental mental control scale

A revised Chinese version that incorporates different cultural contexts of the Parental Mental Control Scale developed by Wang et al. (22, 23) was used in this study. It contains authoritative assertion (e.g., “My parents tell me that what they want me to do is best for me and that I should not have questions about these things;” McDonald’s ω = 0.872), loving withdrawal (e.g., “If I do something my parents don’t like, they will seem cold and unfriendly;” McDonald’s ω = 0.903), and guilt response (e.g., “When I don’t do things the way my parents do, my parents tell me they are disappointed in me;” McDonald’s ω = 0.917), with 18 questions on a 5-point scale ranging from “never” to “daily.” The higher the score, the higher the level of parental psychological control. In this study, the confirmatory factor analysis (CFA) indicators of parental mental control scale were better: χ^2^/df = 3.460, RMSEA = 0.048, RMR = 0.050, AGFI = 0.938, GFI = 0.963, CFI = 0.978. McDonald’s ω for the scale was 0.926.

#### Adolescent problem behavior scale

The Youth Self-Report (YSR), developed by Achenbach and Edelbrock (43) and modified by Liu et al. (44), is a 112-item scale divided into anxiety, depression, withdrawal, somatic complaints, social problems, thinking problems, attention problems, disciplinary behavior, and aggression. In this study, the social problems subscale was used (e.g., “does not get along with other adolescents;” McDonald’s ω = 0.858) and was scored on a 3-point scale, with subjects completing the scale based on their performance over the past 6 months. A score of 0 was assigned for “not acting out,” 1 for “sometimes acting out,” and 2 for “often acting out.” The higher the score, the more serious the social problem. In this study, the confirmatory factor analysis (CFA) indicators of social problems subscale were better: χ^2^/df = 1.101, RMSEA = 0.024, RMR = 0.028, AGFI = 0.963, GFI = 0.979, CFI = 0.989.

#### Emotion regulation scale

The Emotion Regulation Scale was developed by Wang et al. (22, 23) and consists of 14 questions, including expression inhibition (e.g., “When I feel happy, I try not to show it;” McDonald’s ω = 0.808) and reappraisal (e.g., “I try to change my perception of my surroundings to make myself happier;” McDonald’s ω = 0.894). Each dimension has 7 items, including the items that regulated the 5 basic emotions of disgust, anger, sadness, fear, and happiness, and 2 items about whether an individual used a certain strategy in general. Subjects were asked to choose the option that best represented their perceptions on a 7-point rating scale in relation to their actual situation, with 1 representing total disagreement and 7 representing total agreement. Higher scores indicated a stronger degree of that dimension. In this study, the confirmatory factor analysis (CFA) indicators of emotion regulation scale were better: χ^2^/df = 3.990,RMSEA = 0.053, RMR = 0.071, AGFI = 0.957, GFI = 0.981, CFI = 0.978.

### Data analysis

Data were analyzed using IBM SPSS Statistics for Windows, version 22.0 (IBM Corp., Armonk, N.Y., USA). A univariate analysis of variance, Pearson correlation analysis and the Marco PROCESS (Model 4) were used to analyze the relationships among parental psychological control, adolescent emotion regulation and social problems.

## Results

A univariate analysis of variance (UNIANOVA) with number of siblings (only child, non-only child), birth order (firstborn and later born), gender (male and female), family income (≤ 5,000 yuan/month,5,001–10,000 yuan/month, ≥ 10,001 yuan/month) as factor variables, adolescents’ social problems scores as the dependent variable was conducted. There was a significant main effect of number of siblings [*F*(1, 1,144) = 6.180, *p* = 0.013, η_*p*_^2^ = 0.005], and the non-only child adolescents’ social problems score (*M* = 0.377, *SD* = 0.015) was significantly higher than the only child score (*M* = 0.271, *SD* = 0.040). The main effect of family income was close to significant [*F* (2, 1,144) = 2.839, *p* = 0.059, η_*p*_^2^ = 0.005], and the social problems of adolescents with a family income of ≤ 5,000 (*M* = 0. 375, *SD* = 0. 042) were significantly higher than those with family income ≥ 10,001 (*M* = 0. 251, *SD* = 0. 038). There were no significant main effects of birth order [*F* (1, 1,144) = 0.595, *p* = 0.441, η_*p*_^2^ = 0.005] or gender [*F* (1, 1,144) = 1.798, *p* = 0.180, η_*p*_^2^ = 0.002] on adolescents’ social problems scores, nor was there any interaction between them (*ps* > 0.05).

Pearson correlation analysis was conducted to examine relationships among parental psychological control, emotion regulation, and social problems with sibling numbers and family income as covariates. Psychological control was positively related with expression inhibition (*r* = 0.104, *p* < 0.010), psychological control was positively related with social problems (*r* = 0.230, *p* < 0.010), and psychological control was negatively related with cognitive reappraisal (*r* = -0.180, *p* < 0.010). Expression inhibition was positively related with social problems (*r* = 0.211, *p* < 0.010), and cognitive reappraisal was negatively related with social problems (*r* = -0.067, *p* < 0.05) (see Table 1).

**TABLE 1:** Pearson correlation coefficients of the study variables (*N* = 1145).

	*M*	*SD*	1	2	3	4
1. Psychological control	2.584	0.922	–			
2. Expression inhibition	2.810	1.328	0.104**	–		
3. Cognitive reappraisal	3.434	1.369	−0.180**	0.359**	–	
4. Social problems	0.337	0.315	0.230**	0.211**	−0.067*	–

M, mean; SD, standard deviation. **p < 0.010, *p < 0.050.

**FIGURE 2:**
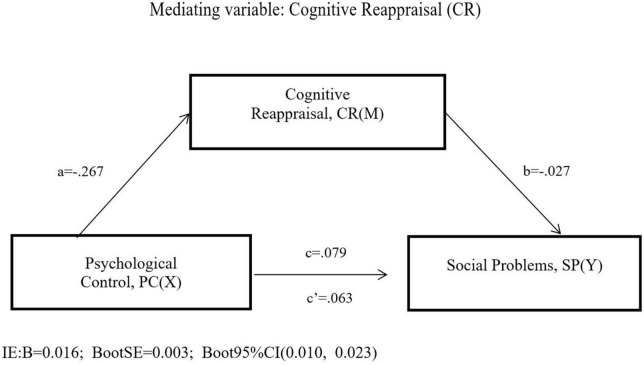
The mediation model of cognitive reappraisal.

Based on the correlation analysis’ results, Model 4 was used to test the mediating effect of cognitive reappraisal on the relation between psychological control and social problems. The results (see in Figure 2 and Table 2) showed that the direct path from psychological control to social problems (β = 0.063, *p* < 0.001) in the absence of cognitive reappraisal was significant. When psychological control and cognitive reappraisal entered the regression equation at the same time, psychological control was significantly associated with cognitive reappraisal (β = -0.267, *p* < 0.001) and social problems (β = 0.079, *p* < 0.001). Cognitive reappraisal significantly predicted social problems (β = -0.027, *p* < 0.001).

**TABLE 2:** Testing the mediation effect of cognitive reappraisal on social problems.

Effects	Path	β	SE	*p*	
Effect PC-CR	a	−0.267	0.043	0.000	
Effect CR-SP	b	−0.027	0.007	0.000	
Total effect PC-SP	c	0.079	0.010	0.000	
Direct effect PC-SP	c′	0.063	0.010	0.000	

PC total effect model (*F* = 63.638; *p* < 0.001; *R*^2^ = 0.053)					

**Indirect effects**	**Path**	**β**	**Boot SE**	**Boot 95%** **CI**	
				
				**LL**	**UL**

Total indirect effect		0.016	0.003	0.010	0.023

**FIGURE 3:**
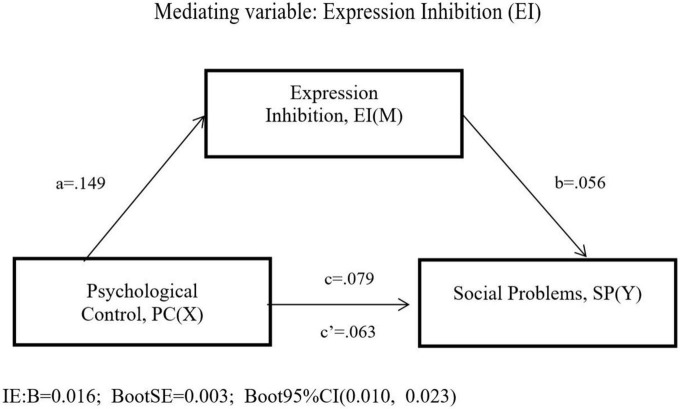
The mediation model of expression inhibition.

Model 4 was used to test the mediating effect of expression inhibition on the relation between psychological control and social problems. The results (see in Figure 3 and Table 3) showed that the direct path from psychological control to social problems (β = 0.063, *p* < 0.001) in the absence of cognitive reappraisal was significant. When psychological control and expression inhibition entered the regression equation at the same time, psychological control was significantly associated with expression inhibition (β = 0.149, *p* < 0.001) and social problems (β = 0.079, *p* < 0.001). Expression inhibition significantly predicted social problems (β = 0.056, *p* < 0.001).

**TABLE 3:** Testing the mediation effect of expression inhibition on social problems.

Effects	Path	β	SE	*p*	
Effect PC-EI	a	0.149	0.042	0.000	
Effect EI-SP	b	0.056	0.007	0.000	
Total effect PC-SP	c	0.079	0.010	0.000	
Direct effect PC-SP	c′	0.063	0.010	0.000	

PC total effect model (*F* = 63.638; *p* < 0.001; *R*^2^ = 0.053)					

**Indirect effects**	**Path**	**β**	**Boot SE**	**Boot 95%** **CI**	
				
				**LL**	**UL**

Total indirect effect		0.016	0.003	0.010	0.023

A bootstrap procedure was applied to assess the size of the indirect effect and confidence intervals. For the indirect effect, 95% bias-corrected accelerated confidence intervals (CIs) without “zero” indicated the significant mediation effect. We generated 5,000 bootstrapping samples. The indirect effects of psychological control on social problems mediated by cognitive reappraisal (ab = 0.007, *SE* = 0.002, 95% CI [0.003, 0.012]) and expression inhibition (ab = 0.008, *SE* = 0.003, 95% CI [0.003, 0.014]) was significant. The mediation effect accounted for 8.917% and 10.191% of the total effect. The 95% confidence interval did not consist of zero, showing that psychological control exerted a significant indirect effect on social problems via cognitive reappraisal and expression inhibition.

## Discussion

In this study, we found that adolescent social problems were influenced by the number of siblings, and adolescents in multi-child families had significantly more social problems than those in one-child families. Consistent with previous studies, only children in adolescence had better social skills than non-only children (45, 46). In a national sample (using data from the 2010 China Household Tracking Survey), Zhang et al. (45) found that with similar personal characteristics, family status, and regional backgrounds, only children in adolescence exhibited a “psychological advantage” in socialization, leading to a subjective belief that they had stronger social competence. However, in actual social situations, there was no significant difference between only children and non-only children from similar home backgrounds (45). From a long-term developmental perspective, social problems of adolescents in multi-child families are temporary. Due to psychological changes in adolescence (self-supporting personality, rebellious psychology, etc.), they begin to alienate and even antagonize their parents and other adults. Compared with one-child families, interpersonal relationships in multi-child families are more complicated, and children with siblings may face more family conflicts (differential treatment by parents, sibling conflicts, etc.) and bear greater psychological pressure, leading to more social problems. However, once they learn to cope with these psychological pressures, they may have better social skills than only children, as sibling interaction is beneficial to the development of interpersonal communication in early adulthood (14, 47).

The study also found that adolescents with a monthly family income below 5,000 yuan had more social problems. This was consistent with the resource dilution hypothesis (4, 5), which emphasizes that the more children in the family, the less the family income and the more likely the family resources are diluted, leading to more social problems among adolescents. The family stress model also posits that family socioeconomic status (SES) will affect the development of children by influencing the family process (48). For example, low family SES will lead to the negative parenting style of parents and eventually hinder the development of children (48). And in our previous studies, we found that mothers of two-child families had higher parenting stress than those of one-child families (13, 49), and in two-child families, families with an income of less than 3,000 yuan had significantly higher maternal stress than families with an income of more than 6,000 yuan (49). Adolescents with lower family SES can obtain and use relatively few kinds of resources, which will make them feel unfairly treated, and cannot adapt to the environment well, thus producing or showing more problem behaviors (50).

In addition, we found that emotion regulation played a partially mediating role in parental psychological control and social problems. On the one hand, parental psychological control could positively predict adolescents’ social problems, indicating that the higher the level of parental psychological control, the more social problems adolescents would have, which was consistent with the research conclusion of Zhang et al. (51). Many empirical studies have also shown that a high level of parental psychological control will lead to more problem behaviors in adolescence (19–21, 24, 28, 29). On the other hand, parental psychological control might affect adolescents’ social problems through emotion regulation: In one case, the more parental psychological control, the more inhibition of emotional expression of adolescents and the more serious social problems (52); in another case, the more parental psychological control, the less cognitive reappraisal of adolescents and the more serious social problems (53). Parents with high levels of psychological control often feel frustrated when their children did not live up to their expectations. Under such circumstances, they tend to blame their children, usually using methods such as making their children feel guilty, expressing disappointment or neglecting their children, or even humiliating their children, all of which result in children’s negative emotional experiences (54). Over time, their children will feel helpless and depressed, which in turn hinders their cognitive and emotional development, leading to more use of negative emotion regulation strategies such as expression inhibition, and less use of positive emotion regulation strategies such as cognitive reappraisal (55). As a result, adolescents will withdraw emotionally or physically, afraid of expressing and transmitting their inner feelings or ideas (56). Further, they may be unwilling to communicate with others, resulting in more social problems and hindering the development of independent consciousness and mental health (57). The partially mediating role of emotion regulation in parental psychological control and social problems can also be explained by problem behavior theory, which holds that family factors such as parental psychological control, individual emotions, and other personality factors jointly affect adolescents’ social problems.

### Implications and limitations

According to the Family System Theory (58), flexible parent-child boundaries are critical for children’s healthy development, and a balance between autonomy and attachment to parents is essential for a child’s healthy adaptation. If parents do not respect their children’s opinions and force their children to meet their own needs, children’s autonomous exploration outside the family will be hindered. Thus, children’s personalized and adaptive development will be suppressed. Traditional Chinese culture emphasizes the authority of elders in the family. To help their children make progress in study, some parents will deliberately be stern and seldom praise their children directly when they achieve success. There are even parents who demean or induce guilt and anxiety in their children, intentionally or unintentionally, to increase their authority. However, parents’ excessive psychological control over their children is not conducive to the healthy growth of adolescents. Therefore, we suggest that parents should adopt a positive parenting style in the process of educating their children. When living with children, parents need to maintain their own independence and leave room for their children to develop such that the children can experience a sense of autonomy. In addition, parents should pay attention to the cultivation of children’s social communication skills, let children learn to use positive emotion regulation to deal with various difficulties, and learn to actively communicate with people when they encounter social problems.

This study had some limitations. Firstly, we focused on adolescent social problems, and relationships among parental psychological control, adolescent emotion regulation, and social problems in China. In the future we will explore whether other aspects of mental health could influence the reported associations with social problems. Secondly, the questionnaire on parental psychological control was answered by adolescents. It is necessary to have the psychological control questions answered by parents to reflect psychological control more comprehensively in our next research work. Thirdly, both problem behavior and emotion regulation were self-reported and lacked objective measurement. In the future, subjective evaluation and actual adolescent social problems should be further clarified. Fourthly, parents’ sibling status and their potential resulting lack of experience in managing negative emotions were not investigated in the study. These should be included as measures in futures studies on the subject. Finally, results from different studies on the effects of parental psychological control on children are often contradictory. Parental psychological control might be less damaging and even beneficial to children’s development in interdependent cultures (59). Therefore, more studies are needed for further validation.

## Data availability statement

The original contributions presented in this study are included in the article/supplementary material, further inquiries can be directed to the corresponding author.

## Ethics statement

The studies involving human participants were reviewed and approved by the Research Ethics Committee of Capital Normal University, Beijing, China. Written informed consent to participate in this study was provided by the participants or their legal guardian/next of kin.

## Author contributions

GQ and YW designed the project and supervised the data collection. GQ, LL, RH, and GD collected and analyzed the data. GQ, YW, WW, CL, XH, AH, YA, and GD wrote the manuscript with input from LL, RL and RH. All authors contributed to the article and approved the submitted version.
